# Estrogen Signaling and the Aging Brain: Context-Dependent Considerations for Postmenopausal Hormone Therapy

**DOI:** 10.1155/2013/814690

**Published:** 2013-07-07

**Authors:** Natasha N. Mott, Toni R. Pak

**Affiliations:** Department of Cell and Molecular Physiology, Loyola University Chicago Stritch School of Medicine, 2160 S First Avenue, Maywood, IL 60153, USA

## Abstract

Recent clinical studies have spurred rigorous debate about the benefits of hormone therapy (HT) for postmenopausal women. Controversy first emerged based on a sharp increase in the risk of cardiovascular disease in participants of the Women's Health Initiative (WHI) studies, suggesting that decades of empirical research in animal models was not necessarily applicable to humans. However, a reexamination of the data from the WHI studies suggests that the timing of HT might be a critical factor and that advanced age and/or length of estrogen deprivation might alter the body's ability to respond to estrogens. Dichotomous estrogenic effects are mediated primarily by the actions of two high-affinity estrogen receptors alpha and beta (ER**α** & ER**β**). The expression of the ERs can be overlapping or distinct, dependent upon brain region, sex, age, and exposure to hormone, and, during the time of menopause, there may be changes in receptor expression profiles, post-translational modifications, and protein:protein interactions that could lead to a completely different environment for E_2_ to exert its effects. In this review, factors affecting estrogen-signaling processes will be discussed with particular attention paid to the expression and transcriptional actions of ER**β** in brain regions that regulate cognition and affect.

## 1. Introduction

According to the CDC (2008), the average lifespan for women in the USA is ~81 years of age. While the average lifespan has been steadily increasing over the past century (~48 years in 1900), the average age at which reproductive senescence, menopause, occurs has remained relatively constant between 45–55 years of age [1, 2]. Including the prepubescent years, this leaves women living about half of their lives without high levels of circulating ovarian hormones. The two primary ovarian hormones are 17*β*-estradiol (E_2_) and progesterone, both of which are required for female reproduction. Many positive anecdotal experiences are reported during times in the reproductive cycle when E_2_ is high, sparking further investigation into the role of E_2_ in various nonreproductive processes, including those pertaining to cognition and mood. The vast majority of basic science studies have described positive effects of E_2_ on cognitive processes at a molecular level, and, importantly, older postmenopausal females exhibit significant deficits when performing tasks that require the use of working memory, attentional processing, and executive function [3–8]. The natural aging process is coincident with menopause, which confounds studies attempting to differentiate between the molecular mechanisms specific to menopause versus aging. Therefore, studies examining the physiological and molecular functions of estrogen receptors during periods of estrogen deprivation with respect to natural aging are requisite to understanding how reintroducing estrogens in aged postmenopausal women will affect neurological processes. In spite of the wealth of studies investigating the effects of HT on relevant health concerns, there are still very few conclusive arguments for or against HT to ameliorate neurological issues. Moreover, it is very likely that the actions of estrogens regulate opposing processes depending upon brain region and genetic composition of neurons involved, creating complex issues regarding the lack of specificity of E_2_ treatment. Nevertheless, some insight into general functions of E_2_ in the brain can be gleaned from existing data that demonstrate that (1) there is a critical window of time surrounding menopause for which HT can be beneficial, suggesting that aging is an important factor, (2) progestins are not likely to be beneficial for cognitive and affective neurological issues, and (3) the type of estrogen used may be crucial. Given these important conclusions, this review will focus on the molecular mechanisms of E_2_ signaling in the brain and how variables that might contribute to these signaling patterns can be altered by age. 

## 2. The Menopausal Transition: E_2_ Decline and Health Concerns

Menopause is defined by the Mayo Clinic as “the permanent end of menstruation and fertility, occurring 12 months after your last menstrual period.” Menopause is marked by a reduced oocyte number attributable to progressive atresia of ovarian follicles and by declining circulating levels of E_2_ and progestins. The perimenopausal transition is typically 4–8 years, during which most women experience symptoms including hot flushes, night sweats, mood swings, sleep disturbances, vaginal dryness and atrophy, as well as urinary incontinence, most of which are alleviated by hormone (E_2_) replacement therapy (HT/ET). Until recently, a great deal of evidence suggested that estrogens have positive effects on cognition, neuroprotection, memory, anxiety, depression, as well as bone and cardiovascular health [5, 9–13]. 

The paramount studies to present negative consequences of HT were the Women's Health Initiative (WHI) and the ancillary studies including the Women's Health Initiative Study on Cognitive Aging (WHISCA) and the Women's Health Initiative Memory Study (WHIMS). Data from these studies showed that a combination therapy of conjugated equine estrogen/medroxyprogesterone acetate (CEE/MPA) increased risk for mild cognitive impairment and decreased global cognitive functioning, but CEE alone did not have any significant effect on cognitive functioning [14–16]. Poststudy analyses have revealed many confounding factors in the WHI studies ranging from the choice of a reference group to the age of participants and the choice of ET used (CEE) [4, 17, 18], as well as the use of MPA, which has been shown to have adverse effects on memory after one dose in adulthood [19]. While the WHI studies showed negative or neutral effects of estrogen therapy, many other basic science and observational studies have shown just the opposite. The Kronos Early Estrogen Prevention Study (KEEPS) recently announced findings that suggested that E_2_ therapy had a positive effect on mood and memory. Participants receiving CEE showed significant improvement in symptoms of depression, anxiety, and a trend toward reduced feelings of anger/hostility. Importantly, CEE treatment or Premarin (Wyeth-Ayerst, Philadelphia, PA, USA) is a mixture of several estrogenic compounds, but primarily estrone sulfate and ring B unsaturated estrogens such as equilin and equilenin, which can differentially activate ER isoforms as compared to E_2_ alone [20]. Participants receiving CEE self-reported a trend toward better recall of printed materials as compared to placebo, and women using transdermal E_2_ tended to report fewer memory-related complaints. Another study performed a meta-analysis of 36 randomized HT clinical trials (RCT) focusing on cognition [21]. The length of treatment, type of memory, variety of hormone, and age of the participant were all variables that drastically altered the outcomes of each trial. Results from the meta-analysis indicated that verbal memory was most often affected by HT, and younger women tended to have a better outcome in this category. There was also a trend toward worse outcomes on memory tests in patients treated with CEE treatment alone compared to those treated with biologically identical E_2_. Moreover, treatment with estrogens alone (i.e., absent cotreatment with progestins) was overall associated with positive results on memory tests. In conclusion, data from these clinical trials have revealed the importance of using bioidentical hormones for HT and that downstream signaling processes for memory and mood can be affected by the choice of estrogen and/or combination of hormones used as therapeutics. 

## 3. Estrogen Receptor Signaling

 Estrogen signaling is mediated primarily through two receptors (ER*α* and ER*β*). ERs are class I members of the nuclear hormone superfamily of receptors, deemed as a ligand-inducible transcription factors [22]. Classically, ERs were thought to be localized in the cytoplasm bound to intracellular chaperone proteins until induced by ligand to translocate to the nucleus, according to the two-step hypothesis coined by Jensen et al. [23]. Following ligand binding, ERs undergo a conformational change that allows for dimerization, translocation to the nucleus, and DNA binding or association with other transcription factors to regulate gene transcription; however, we now know that ER signaling is much more complex.

For example, ERs are involved in other “nongenomic” molecular functions including RNA processing, second-messenger signaling cascades, and rapid dendritic spine formation in neurons. Of particular importance in the brain, the discovery of rapid signaling processes implicates E_2_ as a neuromodulator; however, local synthesis of E_2_ has been the subject of fervent debate. While it is likely that there is *de novo* synthesis of E_2_ within the parenchyma, due to technical challenges, the exact levels and changes with age and circulating hormones have yet to be identified [24, 25]. It is also difficult to determine how local E_2_ may affect ER action. Most reports suggest an implicit role for local E_2_ at the synapse and membrane [26], but whether nuclear/genomic activities of ERs are affected has yet to be established. Recent data from our laboratory demonstrate that E_2_ can alter miRNA-expression [27], and from others have shown that ER*α* can associate with miRNA processing enzymes such as Drosha [28]. Data from our laboratory (unpublished observations) and others have shown that ERs are involved in alternative splicing processes, and one study has demonstrated direct interaction of phosphorylated ER*α* with splicing factor (SF) 3a p120 that potentiates alternative splicing through EGF/E_2_ crosstalk [29]. These relatively novel ER functions may be explained by examining well-studied components of classic NR signaling such as the structural properties of the receptors. 

## 4. Structural Contributions to ER Activity

**Figure 1:**
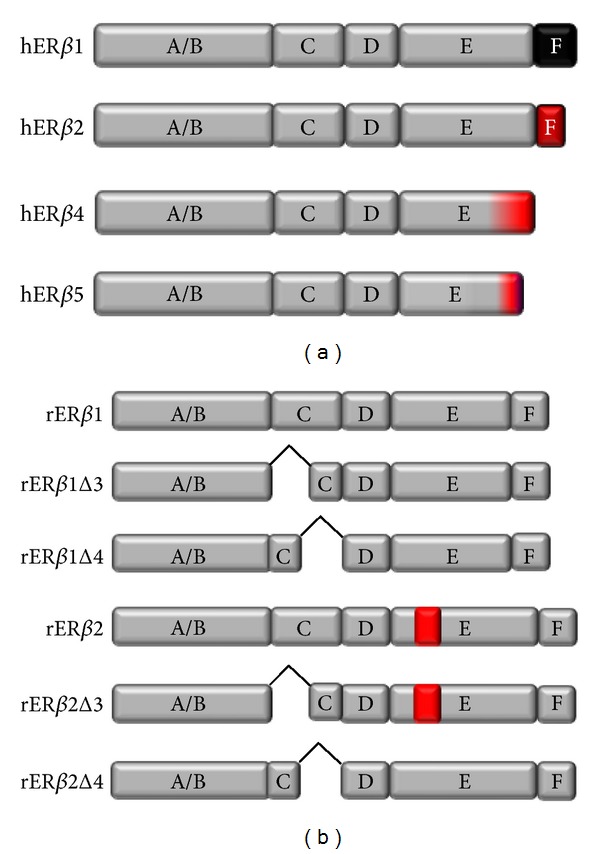
Representative image of domains within human and rat ER*β* splice variants. Human ER*β* splice variants (a) contain truncations and changes in amino acid sequence in the C-terminus E and F domains. Rat ER*β* splice variants (b) contain an 18-amino-acid insert in the LBD/E domain and/or exon 3/4 exclusions in the DNA-binding domain.

Class I nuclear receptors (NRs) including ER*α* and ER*β* have a characteristic structure comprised of five functional domains labeled A–E, and a sixth domain (F) unique to ERs (Figure 1). The A/B domain contains an activator function-1-(AF-1-) like domain that allows for associations with coregulatory proteins and other transcription factors. Notably, the A/B domain is the least conserved domain between ER*α* and ER*β* (17% homology), and it may be responsible for the observed ligand-independent actions of ER*β* [30]. The C domain, is a DNA-binding domain that allows the receptor to bind a specific DNA sequence called an Estrogen Response Element (ERE) to regulate transcription of genes containing this sequence within their promoter region. Two zinc fingers forming a helix-loop-helix structure allow for appropriate spacing (3 nucleotides) between an inverted hexameric palindromic repeat that is described as the canonical ERE. The exact nucleotide sequence of hormone response elements can vary and in part, dictate the affinity an NR has to regulate a particular gene [31]. The D domain is a hinge-like region that allows the receptor to undergo a conformational change once activated and also contains a nuclear localization sequence. The best-studied region of ERs is the E domain, also referred to as the ligand-binding domain (LBD). Characterization using X-ray crystallography has shown that the LBD consists of 12 ordered alpha-helices that are essential for conferring ligand specificity [32]. The orientation of helix 12 is critical to the conformation NRs adopt once bound to a particular type of ligand, and ultimately influence the ability of the receptor to bind other proteins and activate gene transcription. Helix 12 contains the core residues of the activator function-2 (AF-2) domain, a short amphipathic conserved alpha-helix that interacts with coregulatory proteins through an LxxLL motif. Adjacent to the AF-2/E domain is the less characterized F domain that is unique to ERs. ER*α* has a larger F domain than ER*β*, and the two receptors only share about 18% homology within this region. ER*α* dimerization and interactions with coregulators are altered when the F domain is deleted or modified, demonstrating that the F domain is a relevant structure for ER*α* transcriptional regulation, but a clear role for this domain for ER*β* has yet to be determined [33, 34]. Importantly, naturally occurring human ER*β* splice variants have altered E and F domains, which can affect hormone responsiveness in tissues that express these variants.

While the overall sequence homology between ER*α* and ER*β* is greater than 60%, the specific gene targets of each receptor appear to be vastly different. For example, a variety of cancer cell models have identified an antiapoptotic, proliferative role for ER*α*, whereas ER*β* tends to promote apoptosis and regulate antiproliferative genes [35–38]. It is well known that ER*α* and ER*β* are readily able to form heterodimers when expressed in the same cell, adding another layer of complexity to the regulation of estrogen responsive genes. ER*α* and ER*β* both bind EREs, but the affinity for one receptor or the other can depend highly on the specificity of the DNA sequence being regulated and the ligands present [39–41]. Therefore, it is important to consider the overlap in ER*α* and ER*β* preferred transcriptional response elements when both receptors are expressed in the same system. 

## 5. Expression of ERs in the Brain: A Complex Story

**Figure 2:**
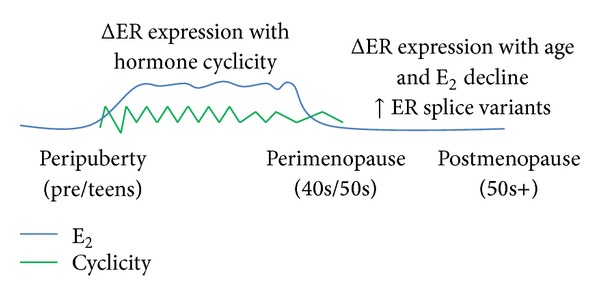
Timeline showing factors affecting ER gene expression throughout the female life-span. Brain ER gene expression patterns are altered with age, sex, and exposure to circulating hormone. Circulating hormones fluctuate with age, most dramatically at the time of puberty and menopause therefore contributing to changes in ER gene expression. Additionally, alternative splicing increases with age, thus potentially diversifying the ER gene expression profile.

The principal determinant of E_2_ action is the expression of ER*α*, ER*β*, their alternatively spliced variants, or some combination of each, which is cell-type specific even within distinct brain nuclei. ER expression has been studied extensively, yet there are few definitive statements that can be made about the regulation of ER*β* expression. It can be noted that ER expression profiles can vary throughout the life-span, in particular when there are dramatic changes in circulating hormone levels, such as puberty and menopause (Figure 2). Not only can ER expression vary dependent upon sex, age, and E_2_ treatment, but these factors can also direct subcellular localization, which ultimately dictates ER functions. Accordingly, contextual studies that map the exact cellular expression patterns of each receptor and their splice variants are a critical first step in creating a comprehensive examination of E_2_-regulated processes in any system.

 The female vertebrate reproductive organs tend to be dominated by the expression of ER*α*, whereas ER*β* is expressed largely in nonreproductive tissues. ER*β* was first cloned from prostate tissue [42], and it has since been shown to have the highest levels of expression in the central nervous system and cardiovascular tissue, as well as lung, kidney, colorectal tissue, mammary tissue, and the immune system [43]. Consequently, some of the most prominent phenotypic problems observed in mice lacking a functional ESR2 gene (*β*ERKO mice) are neurological deficits. By contrast, ER*α* knockout mice have no gross brain-related phenotypes, but they exhibit decreased E_2_-mediated neuroprotection following an ischemic event [44]. Overall, the phenotypes observed in ER*α*- and ER*β*-null mouse models suggest that ER*β* is potentially more important for mediating nonreproductive E_2_-governed processes than ER*α*.

 ER*α* and ER*β* are coexpressed in some regions of the hypothalamus, such as the medial amygdala (MeA), the bed nucleus of the stria terminalis (BNST), and the periaqueductal grey area. However, ER*α* is predominant in hypothalamic nuclei that control reproduction, sexual behavior, and appetite (e.g., arcuate (ARC), medial preoptic (MPoA), and ventromedial (VM)) but ER*β* is the predominant isoform in the nonreproductive associated nuclei (e.g., paraventricular (PVN), supraoptic (SON), and suprachiasmatic (SCN)) as well as the hippocampus, dorsal raphe nuclei, cortex, and cerebellum [45, 46]. In the hippocampus, mRNA and protein for both ERs have been detected and are well established as mediating both genomic and nongenomic processes [47–49]. Nuclear and extranuclear ER*β* mRNA and immunoreactivity (IR) have been detected in principal cells as well as in many other nuclei of cells within the ventral CA2/3 [46, 47]. Although not as prevalent as ER*β*, ER*α* has also been detected in the hippocampus, primarily within GABAergic interneurons [47, 49].

 ER expression is also often found to be sexually dimorphic. As one would expect, many regions of the hypothalamus exhibit a great deal of sexual dimorphism due in part to differences in sexual behavior and regulation of gonadotrophic hormones, but regions such as the BNST also display some sex-related differences in ER expression. For example, ER*α* in the BNST can be induced in somatostatin-positive neurons of male, but not female, rats [50]. ERs have also been shown to be sexually dimorphic in the developing rodent hippocampus, but not in adults [51, 52]. However one report identified ER*β* mRNA in the adult female, but not male, rhesus macaque basal ganglia and hippocampus [53]. Importantly, a lack sexually dimorphic regional ER expression does not preclude differential responses to estrogens, as other effector molecules can alter estrogen-responsive processes.

 Expression of ERs can vary not only with chromosomal sex, but also in response to the hormonal milieu. For instance, it is well accepted that ER*α* expression is autoregulated by E_2_, primarily through proteasomal degradation, [54] but also perhaps on a transcriptional level by E_2_-bound ER*β* [55]. The ER*β* gene (ESR2) promoter region has not been extensively characterized, but it has been shown to contain E_2_-responsive *cis* sequence-binding sites for Oct-1 and Sp-1, which interact with ERs via *trans* factors suggesting a molecular mechanism for E_2_-mediated autoregulation of its receptor. There is also an *Alu* repeat sequence that may contain an ERE that could act as an ER-dependent enhancer [56]. Conversely, *in vitro* and *in vivo* studies investigating the effects of E_2_ on ER*β* expression have yielded inconsistent conclusions depending upon cell type, animal species, and age. For instance, in the T47D human breast cancer cell line, E_2_ upregulated ER*β* [57]. However, ER*β* expression was decreased by E_2_ in mammary glands of lactating mice that coexpress ER*α* [58]. ER*β* was also decreased in the PVN of rats subjected to OVX + E_2_ [59]. Thus, it appears that E_2_ may regulate ER*α* and ER*β*; however, this effect is highly dependent upon cell type, and possibly upon the coexpression of other ERs.

 In addition to sex and E_2_, aging also appears to influence ER expression. Overall, decreased nuclear E_2_ binding has been reported in the hypothalamus and anterior pituitary of aged female rats compared to young ones, but the change in E_2_ binding was not necessarily attributed to a decrease in total ER expression [60, 61], suggesting a shift in the ratio of ERs and/or subcellular localization. While overall nuclear E_2_ binding within the hypothalamus may decrease with age, changes to ER expression patterns with age remain contentious. In general, it appears that age alone does not eliminate ER*α* expression in the brain, but regional specificity and E_2_ availability may be important factors [62, 63], and an increase in ESR promoter methylation has been correlated with age in other systems [64, 65]. One study reported varied middle age-specific reduction in hypothalamic ER with E_2_ treatment [66], yet another study showed that E_2_ decreased hypothalamic ER expression significantly in all ages tested (3, 11, and 20 months) [67]. Specific to ER*α*, a work by Chakraborty and colleagues determined that immunoreactive cell numbers did not always change following OVX and E_2_ replacement. Rather, their study revealed that with advanced age (24–26 months compared to 3-4 and 10–12 months) the number of ER*α*-positive cells was increased or it stayed the same in different hypothalamic nuclei [68]. In the hippocampus, ER*α* was decreased after long-term estrogen deprivation (LTED, 10 weeks), regardless of E_2_ replacement following LTED, but E_2_ deprivation had no effect on ER*β* [11]. The same report demonstrated decreased levels of ER*β* in very old rats (24-month females compared to 3-month diestrus females). In general, most reports suggest that aging decreases ER*β* expression, but, like ER*α*, this effect may be highly region specific. An age-related decrease in ER*β* expression in the brain is underscored by a corresponding increase in CpG methylation of the ESR2 promoter in middle-aged (9–12 months) rats [69]. Other reports describe decreases in ER*β* protein and message in some areas but not in others [63, 70]. Taken together, there are a number of reports attempting to identify the parameters that control ER expression such as age, sex, and response to E_2_; however, with such vast deviations in expression with cell type there is still much to be learned about expression of these receptors, especially in brain regions controlling nonreproductive behaviors.

## 6. Alternative Splice Variants

 Based upon the highly variable reports that differ in sex and age of animals as well as exposure to hormone, it may be possible that these studies are unknowingly detecting changes in splice variant expression, which could change E_2_ responsiveness as well as downstream gene regulation. Not only can ERs heterodimerize to regulate gene transcription, but there are a number of alternatively spliced variants of each receptor that are endogenously expressed and that potentially contribute to the diverse tissue-specific actions of E_2_. Alternative splicing of ERs alters inherent signaling properties of the receptor including ligand, and DNA-binding affinities, nuclear localization, and dimerization, depending on where the alternative splice site is encoded. A number of ER splice variant transcripts and other proteins have been identified in demented human brains, breast, and prostate, and, in some reports, an increase in alternative splicing is correlated with pathology [71–75]. Also interesting, age alone may increase alternative splicing of some gene products [76]. The identified ER*β* human splice variants are truncated at the C-terminus of the receptor (Figure 1(a)); however, we provided experimental evidence that the C-terminus of the receptor is not required for ER*β*-mediated transcription, especially with regard to the identified human splice variants [77]. Unlike the human splice variants, rodent ER*β* splice variants identified to date have been shown to have either an exon inclusion in the ligand-binding domain, creating (rER*β*2), or an exon deletion in the DNA-binding domain rER*β*1Δ3 or rER*β*1Δ4 or both rER*β*2Δ3 and rER*β*2Δ4 (Figure 1(b)) [37, 78, 79]. Exon inclusion (rER*β*2 variants) has been shown to produce a protein that binds E_2_ with a 35-fold decrease in affinity. In contrast, ERs with exon 3 and 4 deletions are unable to bind DNA, but they can still mediate transcription through protein:protein interactions with other transcription factors such as AP-1, and it can bind E_2_ as well as rER*β*1 [37, 80]. Importantly, the transcriptional functions of rER*β*1 are significantly altered when coexpressed with other splice variants, likely due to a weaker interaction with coactivator proteins [81, 82]. Despite lower E_2_ binding and/or lack of DNA binding, the rodent and human splice variants retain a constitutive ligand-independent transcriptional function, at both basic and complex promoters [77, 83, 84], suggesting that these splice variants have an important endogenous biological function. Indeed, unliganded ER*β*1 or apo-ER*β*1 has been reported to regulate a subset of genes distinct from those regulated by ER*β*1 when bound to E_2_ [41]. Conversely, the human splice variants do not bind ligand with great affinity [85], and they might therefore only regulate the class of genes that unliganded ER*β* targets. 

The downstream target genes of ER*β* splice variants might be an important consideration at the time of menopause, as ER expression profiles and alternative splicing tend to change with age [76]. One recent report demonstrated an increase in ER*β*2 expression in the hippocampus of 9-month old, middle-aged rats following short-term (6 days) E_2_ deprivation that was significantly decreased compared to the Sham group after E_2_ administration [86]. Importantly, E_2_ replacement no longer affected ER*β*2 expression in the hippocampus after LTED (180 days). That study also reported a decrease in hippocampal neurogenesis and increased floating behavior in a forced swim test, thus functionally correlating increased ER*β*2 with mood regulation and potentially cognition. Thus, the expression and functions of ER*β* splice variants are absolutely critical to understand the effects of estrogen particularly at times of sustained E_2_ deprivation with regard to cognition and affect. While ER*β*2 expression has been assessed in the young male rat brain [87], and other variants have been described in some brain regions [80, 88], there is a general lack of data on most ER*β* splice variants, especially in aged female brains. 

Some of the splice variants identified to date have been characterized as dominant negative receptors, serving to inhibit activation of the full-length receptor [89]; however, most identified variants do not bind ligand with the same affinity and have the potential to differentially regulate target genes. While several splice variants for ER*β* have been identified in many model systems including mouse [90], rat [45, 46], and monkey [91], there is a general lack of comparative studies on expression and functionality of human ER*β* variants, especially in neuronal systems. Furthermore, changing expression levels of one or more alternatively spliced variants during a period of E_2_ deprivation may drastically change general receptivity and downstream functions of E_2._

## 7. Novel Protein:Protein Interactions for E_2_-Mediated Nuclear Processes

Protein:protein interactions are an essential relay in the regulation of dynamic cellular processes. Immediately following translation, ERs typically associate with a chaperone protein to ensure proper folding, protect from degradation, and assist the ER in becoming poised to accept ligand. Once bound to ligand, ERs can dimerize and act as transcription factors to mediate gene regulation or associate with membrane proteins to initiate a signaling cascade. When acting as transcription factors, ERs associate with a number of coregulatory proteins that assist in activating or repressing E_2_-regulated genes. Coregulatory interactions are more characterized for ER*α* than ER*β*, and, importantly, less clear is how ER*β* mediates ligand-independent transcription. In addition to the well-established ER interaction partners, many novel interacting proteins have not yet been characterized and could be critical for nuclear processes not limited to gene transcription.

## 8. HSPs and Chaperone Proteins

 According to the classical two-step hypothesis, inactive nuclear receptors are constantly accompanied and protected from degradation by a number of chaperone proteins, typically members of the heat-shock protein (HSP) family. This receptor:chaperone complex has been studied extensively, and while the idea of a protective role for chaperones is well supported, this complex can also perform other functions. For instance, HSP:ER complexes can serve to preactivate a hormone receptor by forcing a conformational change in ER such that it is able to bind its cognate hormone. The initial HSP complex consists of the ER, HSP70, and HSP70-interacting protein (HiP), as well as other accessory and scaffolding proteins [92]. HSP90 is recruited to the complex, and HSP70 dissociates, creating the mature HSP:ER complex [93]. HSP90 induces a conformational change in the nuclear receptor, and the ER is released from the complex, ready to dimerize and bind DNA or other transcription factors to regulate gene transcription. However, some studies suggest that HSPs could have a broader role than originally thought. For example, in Drosophila, HSPs are required for DNA binding, and in some instances they may regulate NR action [94]. Interestingly, aging and E_2_ can alter HSP70 in a cell-type specific manner [95–98]. However, recent data from our lab (Table 1) demonstrated that HSP70 more readily associates with ER*β* in aged female hippocampus following E_2_ treatment compared to the young ones in which HSP70:ER*β* association decreased following E_2_ treatment. We also observed no significant changes in HSP70 or ER*β* expression, suggesting that changes in the HSP70:ER*β* interaction with age in response to E_2_ change are a result of E_2_ responsiveness and/or activation of ER*β*.

## 9. Transcriptional Proteins and ERs

The process of transcribing DNA into RNA is a systematic process that involves multiprotein complexes binding to DNA, modifying histone marks, and initiating RNA synthesis. ER*α*, but not ER*β*, has been shown to directly interact with TFIIB, IIE, IIF, and TIID proteins that initiate transcription [99, 100]. However, experimental evidence from co-immunoprecipitation studies has demonstrated interactions between ER*β* coregulatory proteins as well as other transcription factors. Coregulatory proteins are transcriptional accessory proteins that enhance or repress transcription of target genes. In general, coactivators enhance gene transcription, whereas corepressors block it. However, recent data suggest that seemingly nontranscriptional proteins may have context-dependent coregulatory functions. Importantly, certain coregulators can also be governed by age and E_2_ [101–103]; thus, recent discoveries imply that ER-mediated gene regulation is not as well understood as previously thought.

The best studied and well-established group of coregulatory proteins that selectively associate with NRs is the steroid receptor coactivator (SRC/p160) family. The SRC family is composed of three members, SRC-1, SRC-2, and SRC-3, all of which contain canonical LxxLL motifs known as the nuclear receptor (NR) box. This motif interacts with AF-2 domains in ER*β*, as well as other NR family members such as glucocorticoid receptor (GR), progesterone receptor (PR), thyroid hormone receptor (TR), and ER*α* [104]. SRC members have intrinsic histone acetyltransferase activity (HAT, DNA activating) and interact with CREB-binding protein (CBP) [105]. CBP/p300 proteins are also coactivators that have intrinsic HAT activity and can recruit ASC-2 and other known coregulatory proteins [106]. Confirmed coregulatory interaction partners for several NRs that do not belong to the SRC family include estrogen-receptor-association protein (ERAP 140) [107], nuclear corepressor (NCoR) [108] silencing mediator of retinoic acid and thyroid hormone receptor (SMRT) [109], and many others. As is the case with our understanding of ER*β* interactions with basic transcriptional machinery, studies investigating ER*β*:coregulator interactions are sparse, which may be due to uniquely challenging issues associated with ER*β*, such as a lack of high-fidelity biochemical tools, complicated structural properties, and, or pleiotropic physiological actions that are specific to ER*β*.

In 2010, Anna Ma lovannaya and colleagues directed a high-throughput study (not including ER*β*) aiming at compiling a database for the endogenous coregulator pool “nuclear receptor complexome” [110]. In this study, a number of novel protein interactions were identified, and studies such as these are identifying proteins as “coregulators” that had been previously thought to serve completely different functions. One group of relatively novel coregulatory proteins are the E3 ubiquitin-protein ligases such as E6-associated proteins (E6-AP) [111]. While these proteins were thought to serve primarily as ubiquitin-conjugating enzymes, they have recently been highlighted as transcriptional enhancers of NR-mediated activity independent of ligase function. Similarly, a group of E3-ligases that conjugate small ubiquitin like modifier (SUMO) proteins to a target protein called PIAS are also now considered NR coregulators and they utilize a typical LxxLL motif. In one study, a decrease in ER expression following LTED or with advanced age coincided with an increase in ER association with an E3-ubiquitin ligase, CHIP [11]. Together, these newly described roles for HSPs and E3 ligases raise novel questions about estrogen signaling, such as when is an E3-ligase:ER complex targeted for transcriptional regulation versus degradation? Also, when are HSPs merely performing a chaperone/protective function versus directing transcriptional processes? Future efforts aiming at elucidating the complexity of age-related changes in receptor structure and recruitment of coregulatory proteins could provide important insight into these seemingly paradoxical findings.

## 10. Nuclear Actin: Setting the Stage

Coregulatory interactions may be poised upon a bed of nuclear actin, which has recently been identified as a dynamic molecular stage for which many nuclear processes are performed such as transcription, chromatin remodeling, mRNA processing, and nuclear import/export. The general events that initiate transcription are well established; however, the process by which all of the molecular components are temporally layered into a complex is still unclear. Nuclear actin is essential in forming the preinitiation complex on a promoter, elongation, and RNP organization, as well as remodeling of chromatin [112–114], and, as mentioned previously, ERs are also key factors in these processes. In one study, ER*α* and *β*-actin were coimmunoprecipitated on the E_2_ responsive pS2/*TFF*1 promoter, indicating that ER and nuclear actin may work in concert to regulate transcriptional processes under control of estrogens [115]. The interaction between ERs and actin is not yet fully investigated, but data from our lab (unpublished observations) and others [116] imply that both ER*α* and ER*β* may utilize nuclear actin to perform various functions. Another actin-binding, protein gelsolin, caps actin filament ends, and it has been shown to be an NR coactivator [117, 118]. Gelsolin may assist in actin polymerization, allowing transcriptional machinery to be brought in proximity of target genes; however, it remains unclear how gelsolin enhances AR/ER transcriptional activity. Data from our lab indicate that gelsolin:ER*β* interactions increase with E_2_ treatment in young but not aged animals (Table 1). Gelsolin has been shown to increase with age [119], but a lack of significant interaction with ER*β* despite increased expression of gelsolin could again suggest an alteration in ER*β* function with age. 

 Actin is also commonly associated with ubiquitous multifunctional RNA-binding proteins such as heterologous nuclear riboproteins (hnRNPs), which also associate with ERs [120]. hnRNPs associate within the matrix of nuclear actin, accompany transcripts out of the nucleus, participate in alternative splicing, and can modulate transcription [121]. Phosphorylated hnRNPK has been shown to mediate translation of specific mRNAs [122], and hnRNPH is involved in splicing and mRNA polyadenylation [123, 124]. In the past, the association of NRs with hnRNPs was thought to be nonspecific due to the ubiquitous nature of these proteins, but recent studies are no longer ruling out an important interaction between NRs and hnRNPs that may assist in transcription and/or splicing [125, 126]. Data from our lab and others demonstrate a dynamic interaction between both ER*α* and ER*β* and hnRNPs (Table 1), and, furthermore, data, demonstrated that E_2_ might regulate expression of members of the hnRNP family [127]. As noted previously, age-related increases in splicing could lead to aberrant signaling, not only for E_2_-mediated processes, but also for cellular processes in general.

**Figure 3:**
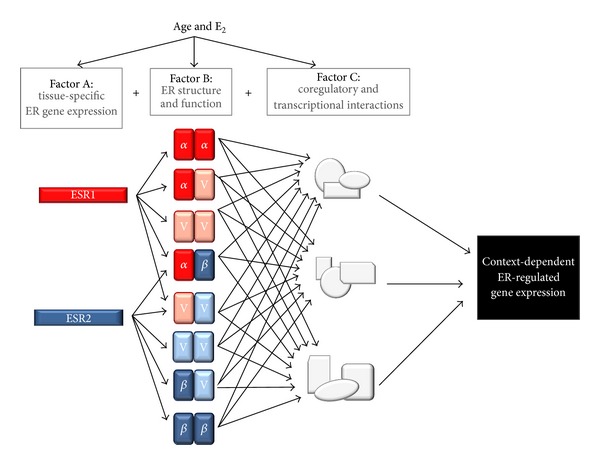
Age and hormonal milieu exponentially increase the potential diversity of estrogen receptor signaling leading to context-dependent gene regulation. Age and E_2_ influence ER gene expression, alternative splicing, coregulatory protein expression, and interaction, which ultimately direct ER-target gene transcription.

 Nuclear ER interaction partners have historically been a distinct class of nuclear receptor coregulators that seemed to solely assist ERs in gene transcription; however, the number of interaction partners for ERs is increasing. Further investigation into ER*β*-associated proteins is required, as far as NRs are concerned; data specific to ER*β* are inadequate to make broad conclusions. Moreover, post-translational modifications to coregulatory proteins, ERs or changes in their expression patterns due to age or sustained estrogen deprivation could all contribute to an altered microenvironment, setting the stage for atypical estrogen signaling upon therapeutic reinstatement of hormones (Figure 3).

## 11. Estrogens and Cognition 

Most empirical and observational data give merit to the idea that estrogens have a positive effect on cognitive processes, increased spine densities [128, 129], enhanced synaptic plasticity [130–132], and improved memory [133, 134]; however, the particular receptor(s) and the mechanisms that regulate these processes remain unclear. There are a myriad of behavioral studies suggesting that E_2_ enhances prefrontal cortex (PFC) and hippocampal-dependent tasks. For example, long-term E_2_ deprivation diminished aged female rhesus macaques' performance in a delayed response task, a PFC- dependent task [135]. E_2_ also enhanced object recognition under a number of different paradigms [136–138], and there are also multiple lines of evidence supporting E_2_-mediated neuroprotection which may be important for cognition, especially after stroke [139–142]. 

Pharmacological targeting of the receptors with ER selective ligands has been a standard method for investigating the behavioral, physiological, and cellular actions of E_2_ mediated distinctly through ER*α* and/or ER*β*; however, valuable insight has also come from the ER*β*-null (*β*ERKO) mice. *β*ERKO mice have significantly fewer neurons in the cortex, hypothalamus, amygdala, and ventral tegmental area compared to WT. They also exhibit neuronal shrinkage and hyperproliferation of glia by 3 months of age, as well as having high levels of apoE and apoE-dependent deposition of amyloid plaques throughout the CNS by 12 months of age [143]. These mice also demonstrate spatial learning deficits in the Morris water maze [144] and a decrease in hippocampal- and amygdala-dependent memory in a fear-conditioning paradigm that is accompanied by decreased synaptic plasticity in hippocampal slice preparations [145]. The critical role of ER*β* in higher-level brain functions has been deduced from these studies and others, warranting a full investigation of the wide-spread molecular actions of E_2_ known to contribute to cellular processes on at least two levels: at the synapse and on the genome. 

Long-term potentiation (LTP) is an important component of learning and memory. It represents an increase in synaptic transmission and plasticity that underlies cognitive behaviors, and it is readily altered by E_2_ in many circumstances. In fact, application of an aromatase inhibitor eliminates CA LTP generated by theta-burst stimulation in intact female neurons, but not male or OVX animals, posing a potentially serious concern for women using aromatase inhibitors for therapeutic treatment of breast cancer [146]. E_2_ can also enhance or suppress long-term depression (LTD), reducing synaptic transmission, which may be dependent upon the specific receptors involved. In aged male CA1 cells, E_2_ decreased LTD [147]; however, E_2_ enhanced LTP in the cerebellum where ER*β* is the predominately expressed cognate receptor [148]. 

 Although the majority of studies on cognitive process focus on the rapid effects of E_2_, late-phase long-term potentiation (L-LTP), depends upon transcription and translation of new mRNA [149] to sustain an increase in synaptic transmission. E_2_ has been shown to regulate LTP in CA1 pyramidal cells [150] over the span of 48 hours, and this regulation appears to be dependent upon a higher ratio of NMDAR relative to AMPAR. LTP induction requires activation of NR2A-containing NMDARs; however, increased expression of NR2B potentiates LTP magnitude [151]. Notably, E_2_ increased expression of NR2B mRNA and NR2B expression at the synapse [152, 153], and the E_2_-induced increase in LTP can be abolished by blocking NR2B receptors [154], suggesting a transcriptional role for ERs in synaptic plasticity. Moreover, E_2_ application may increase CREB expression and the amount of phosphorylated CREB in regions such as the amygdala [155] and BNST [117, 155], which may be critical in the formation of long-term memories. Taken together, these data demonstrate that E_2_ regulates neuronal plasticity and memory through its original role as a transcription factor, and also by acting as a general intracellular signaling molecule through regulation of NMDARs and CREB. However, to date, there are little data on the mechanisms by which ER*β* regulates these processes, or how the same principles of plasticity may apply to other neurological issues. 

## 12. Estrogens and Mood Regulation 

A range of behavioral experiments indicate that E_2_ modulation of stress, mood, and affect is a complex story, with considerable conflicting data that may, as in other processes, be explained in part by distinct roles for ER*α* and ER*β*. Anecdotally, many women report mood fluctuations as corresponding to changes in circulating estrogen levels, such as what occurs during the menstrual cycle, peripuberty, postpartum, and peri/postmenopause. Incidence of anxiety and depression are observed at perimenopause and when hormone levels are fluctuating [156, 157]. However, E_2_ can also exhibit anxiogenic properties, and often anxiety and depression present in a comorbid fashion, especially in women [158, 159]. Interestingly, after the age of 55, bouts of depression and anxiety appear to decrease in women [160]. As previously mentioned, perimenopausal women receiving CEE in the KEEPs study reported an improvement in mood, and the primary actions of CEE tend to be mediated through ER*β* [20]. A plethora of behavioral studies has mounted in response to observational reports, and at first glance it appears that ER*β* has an anxiolytic and antidepressive role; however, there is still an immense void to be filled with respect to biochemical and molecular mechanisms of ER*β* and affective disorders. Elucidating the precise molecular mechanisms that require ER*β* in plasticity and neurotransmitter processing in brain regions regulating these behaviors will help clarify the role of E_2_ in stress- and mood-related processes.

Contemporary hypotheses concerning the onset of affective disorders revolve around perturbations to the central processing of environmental stress. The hypothalamic-pituitary-adrenal (HPA) axis is the 3-tiered hierarchical biological system that mediates physical or psychological response to stressors. The primary steroid regulating the HPA axis is cortisol/corticosterone (humans/rats, CORT), a glucocorticoid receptor (GR) ligand that is produced from the adrenals to exert negative feedback upon the HPA system to effectively modulate response to stressors. The paraventricular nucleus of hypothalamus (PVN) produces two neuropeptides, corticotropin-releasing hormone (CRH) and arginine vasopressin (AVP), to activate the HPA axis. CRF and AVP synergistically stimulate release of adrenocorticotropic hormone (ACTH) from the anterior pituitary, which acts on the adrenal cortex to produce CORT. CORT binds GR and negatively regulates CRF and AVP expressions and releases through classical negative feedback mechanisms [161, 162]. ER*β* is the main ER expressed in the PVN [158, 163–165], and regulation of AVP is an interesting example of how ER action can vary. AVP expression fluctuates during the menstrual cycle and is usually highest when E_2_ is low. In fact, oral contraceptives appear to decrease AVP expression, and E_2_ is thought to inhibit AVP in the human SON [166]. In the rodent system, ER*β* and its splice variants activate the rodent AVP promoter independent of ligand [84]; however, the human promoter is repressed by ER*β* and splice variants. This discrepancy between the human and rat was mediated by an AP-1 response element on the human AVP promoter that is not present in the rat. Importantly, ER*β* acted similarly in the two systems when the AP-1 sequence was deleted from the human promoter, underscoring the striking alterations that small changes in DNA sequence can invoke in E_2_ signaling pathways and the importance of understanding the experimental context upon which such conclusions are based [77]. On the contrary, rat and human CRF expression was increased in response to E_2_ in rodent, monkey, and human hypothalamus, but it was inhibited in the placenta [167–170].

In addition to AVP and CRF, glutamatergic and GABAergic projects from regions like the BNST, AMY, PFC, and hippocampus all express ER*β* [45, 46] and are likely targets for E_2_ to exert effects on the HPA axis. Moreover, decreased ER*β* mRNA in postmortem locus coeruleus has been found to correlate with suicide [13], and, even more recently, ER*β*-mediated hippocampal nitric oxide levels have been implicated in affective behaviors in females, but not males [171]. Neurotransmitter release from these regions influences mood, affect, and stress responses, and E_2_ increases the rate of monoamine oxidase degradation and serotonin transport which enhances serotonin at the synapse; E_2_ also increases serotonin receptor expression [172, 173]. Dopamine and serotonin [174] are diminished in the BNST, POA, and hippocampus and caudate putamen (dopamine) of *β*ERKO mice [174] further implicating an important role for ER*β* in the regulation of emotion and mood. *β*ERKO mice also display serious morphological and functional abnormalities in the brain that correlate to increased depression and anxiety [12, 175–178]. In addition to *β*ERKO studies, administration of ER*β* selective agonists (diarylpoprionitrile, DPN) decreases both stress markers and anxiety-related behaviors in rats [158]. In fact, there have been several studies implicating ER*β* and its variants in affective behaviors, but the molecular mechanisms remain poorly understood. 

## 13. Summary

Estrogen-receptor-mediated signaling in the brain regulates neurological processes, many of which translate to cognitive and affective behavioral outputs. When estrogen is declining and becomes replete, as in menopause, a number of neurophysiological changes occur, producing some unwanted changes. The most common and logical remedy is replacement of bioidentical hormone, E_2_; however, this treatment can be problematic depending upon the length of time a woman has been in a postmenopausal, estrogen-deprived state. This suggests that there is a molecular switch in estrogen-mediated signaling that may allow for drastic change in ER signaling, not to mention the interaction of E_2_ signaling components and the natural aging process. These changes are likely to include alterations to receptor profiles including expression of alternatively spliced variants that respond differently to E_2_, changes in the cellular microenvironment that can alter the protein:protein associations which ultimately leads to changes in ER-mediated gene transcription, and synaptic transmission. ER*β* in particular is widely expressed and implicated positively in the regulation of memory and mood fluctuations, two of the most commonly reported neurological issues in postmenopausal women. It is important to understand the actions of ER*β* in the areas regulating these processes to identify what, when, how, and for whom hormone therapy may be a useful treatment to rectify cognitive and affective issues.

## Figures and Tables

**Table 1:** Protein interactions with ER*β* were altered by age and *E*_2_. Selected proteins that were significantly altered (*P* < 0.05) in their association with ER*β* depending on age and *E*_2_ treatment. Experimental paradigm: young (3 month) and aged (18 month) female Fischer 344 rats were ovariectomized and hormone deprived for 7 days. Following deprivation, animals were administered 2.5 *μ*g/kg *E*_2_ (plasma levels = 79.45 ± 22.5 pg/mL) or vehicle (safflower oil) via subcutaneous injection once/day for 3 days. Nuclear protein was isolated from the ventral hippocampus and coimmunoprecipitated for ER*β* (a beam 14C8) and associated proteins. Protein interactions were identified and quantified using 2D-DIGE/DeCyder and ESI MS/MS. YV = young + vehicle; YE = young + *E*_2_; AV = aged + vehicle; AE = aged + *E*_2_.

Accession no.	Molecular weight (Kda)	Estimated isoelectric point	PEAKS score	% Coverage	ID	Interaction with ER*β*				Function
						Young vehicle	Young *E*_2_	Aged vehicle	Aged *E*_2_	
gi∣ 149038929	80	5.75	49.4	6.43	Gelsolin	—	↑	—	—	Actin-binding coactivator
gi∣ 116242507	75	5.97	93	14.58	Heat-shock protein 70	—	↓	—	↑	Chaperone
gi∣ 120538378	47	5.7	93.2	10.72	Heterogeneous nuclear ribonucleoprotein H1/2	—	↑	—	—	RNA splicing
